# A Robust Noise Estimation Algorithm Based on Redundant Prediction and Local Statistics

**DOI:** 10.3390/s24010168

**Published:** 2023-12-28

**Authors:** Huangxin Xie, Shengxian Yi, Zhongjiong Yang

**Affiliations:** 1 State Key Laboratory of High-Performance Complex Manufacturing, School of Mechanical and Electrical Engineering, Central South University, Changsha 410083, China; xhx981014@126.com; 2 School of Mechanical and Electrical Engineering, Changsha University, Changsha 410022, China; shengxian202310@163.com

**Keywords:** homogenous patches, median absolute deviation, noise estimation, nonlinear regression, redundant dimensions

## Abstract

Blind noise level estimation is a key issue in image processing applications that helps improve the visualization and perceptual quality of images. In this paper, we propose an improved block-based noise level estimation algorithm. The proposed algorithm first extracts homogenous patches from a single noisy image using local features, obtaining the covariance matrix eigenvalues of the patches, and constructs dynamic thresholds for outlier discrimination. By analyzing the correlations between scene complexity, noise strength, and other parameters, a nonlinear discriminant coefficient regression model is fitted to accurately predict the number of redundant dimensions and calculate the actual noise level according to the statistical properties of the elements in the redundancy dimension. The experimental results show that the accuracy and robustness of the proposed algorithm are better than those of the existing noise estimation algorithms in various scenes under different noise levels. It performs well overall in terms of performance and execution speed.

## 1. Introduction

With the wide application of digital images in various fields, image quality requirements are increasing. However, images are affected by various disturbances during acquisition, transmission, and processing. Different levels of noise appear during these processes; this noise not only reduces the image quality but also affects the subsequent image processing and analysis. Image noise estimation aims to obtain the accurate noise standard deviation and use it as a priori information to select appropriate algorithm parameters for subsequent image processing activities such as image denoising [1,2,3,4,5], restoration [6], compression [7,8], and segmentation [9]. The accuracy of the estimation results will directly determine the performance of the algorithm.

### 1.1. Related Works

Additive Gaussian White Noise (AWGN), as the most common type of noise in image processing, is the most widely used noise model and research object. In recent decades, the estimation of AWGN in digital images has been widely studied. The existing noise estimation methods can be roughly categorized into statistical approaches, transform domain-based approaches, filter-based approaches, and patch-based approaches. For some specific application scenarios or noise types, the noise in an image can be assumed to follow a specific probability distribution, and the corresponding noise model [10,11] can be constructed to compute the noise level using statistical information, such as the local variance [12] and histogram [13], of the image itself. Zoran et al. [14] exploited the fact that the kurtosis value in the natural noise-free image has a scale-invariant property, related the noise variance to the kurtosis, and transformed the noise level estimation problem into a nonlinear optimization problem. Chen et al. [15] used statistical measures to describe the distribution of pixel values and constructed corresponding noise models. Maximum likelihood estimation is often used to estimate the parameters of a noise distribution, leading to a more accurate understanding of the properties of the noise [16]. However, the kurtosis scale invariance assumption has limitations for images with highly directional edges or large smooth regions [17]. Statistical approaches are more suitable for simple noise models, but they rely on the a priori information of the noise model and have limited adaptability to different scenes. These approaches may not be able to estimate the noise efficiently for different types of images and noise.

Transform domain methods usually transform the image to other domains through discrete cosine transform or wavelet transform to separate the noise from the image information in the transform domain [18,19]. Liu et al. [20] proposed a method based on the singular value decomposition transform, which uses the tail values of the singular value sequence to approximate the value of the image noise level. Khalilh et al. [21] proposed using the wavelet-transformed coefficients of the details of most of the subbands to reflect only the noise components. Hashemi et al. [22] estimated the noise variance using posterior distribution by establishing the prior probability distribution of the noise variance using the Bayesian inference method. However, the domain change may introduce errors in the image representation and a loss of signal information [23], and these methods are not satisfactory for working with nonsmooth noise or high-frequency image signals.

Filter-based approaches mainly consist of applying an appropriate filter to the image to suppress the high-frequency noise components. The noise estimation results are obtained indirectly by analyzing the image before and after filtering. Immerkar et al. [24] proposed a weighted local mean method to obtain an unbiased estimation of the noise variance by averaging and differencing the image using a matrix mask and estimating the noise variance using polynomial approximation. Olivier et al. [25] proposed a noise estimator based on a step-signal model that used polarization derivatives, directional derivatives, and a nonlinear combination of these derivatives to estimate the noise distribution. Edge detectors such as the Laplace operator are used in these approaches to suppress the structural information of the image and to ensure that the structure and detail do not interfere with the estimation of the noise variance [26]. However, some image structure is always present in the filtered image, and the difference between the filtered image and the original image cannot immediately and directly be assumed to be noise. Such methods can also introduce distortions and are limited by the image structure information and noise characteristics, and their accuracy is significantly reduced for images with rich textures.

Patch-based approaches generally decompose the noisy image into a set of patches and select homogenous patches using pixel brightness proximity and simple texture and structure as selection criteria. Assuming that the pixel value variations of homogenous patches are mainly caused by noise, principal component analysis (PCA) is often used to separate the noise component, and local statistical properties are used to estimate the global noise level. Liu et al. [27] iteratively searched the image for weak-textured patches using the gradient values and statistics of the image patches. Pyatykh et al. [28] used principal component analysis to estimate the noise level of an image. By analyzing the image patch covariance matrix, the principal components containing noise were identified to obtain an accurate noise estimation. Fang et al. [29] proposed a new patch-based algorithm that linearly combines overestimated and underestimated results to yield higher accuracy and robustness. Hou et al. [30] introduced a pixel-level nonlocal self-similarity prior to searching for similar pixels to accurately and quickly estimate the noise level. Ping et al. [31] used image self-similarity and separability to decompose an image into noise principal components and detail components. They then used the principal texture representation to estimate the noise principal components and compute the principal component via image texture variance. Dutta et al. [32,33] used quantum mechanical concepts to represent and process signals and images, proposing novel algorithms and frameworks that bring new ideas and approaches to the field of image noise research.

The advantage of patch-based approaches is that they can fully exploit the statistical features of the local region and reflect the global information with local features. Unlike traditional transform domains and statistical approaches, they can adapt to different noise distributions and scene structures. They can also be combined with air domain and frequency domain processing tools [34,35], providing greater flexibility and applicability. However, the method also has some challenges, such as selecting the appropriate patch size and image coverage and reducing the interference of the original image structure information in the noise estimation.

### 1.2. Motivations and Contributions

This study focuses on the above problems, analyses the relationship between the eigenvalues of homogenous patches and the actual noise level, and designs parameters and strategies that can be adaptively adjusted according to the specific situation in order to obtain accurate and reliable noise estimation results. 

In particular, the main contributions of this paper can be summarized as follows:

- We present the limitations of the traditional method of homogenous patch selection, analyze the effect of the eigenvalues of uniform blocks on the noise estimation results, and provide empirical evidence and a theoretical basis for the phenomena of underestimation and overestimation.
- We use the median absolute deviation (MAD) to remove the eigenvalues of the main dimension and predict the number of redundant dimensions. Through statistical analysis of the correlation between the adaptive discriminant coefficients and the input variables, we established a multivariate nonlinear regression model.
- We show various experimental results on complex benchmark datasets. These results demonstrate that the proposed algorithm performs well in a variety of scenarios with different noise conditions.

### 1.3. Paper Organization

The rest of this paper is organized as follows: Section 2 briefly overviews the noise estimation method based on principal component analysis and analyzes the relationship between the minimum eigenvalue and the actual noise level. Section 3 describes the proposed method in detail. Section 4 specifies the parameter settings, reports the experimental results of the proposed method on a benchmark dataset, and validates its effectiveness by comparing its performance with that of state-of-the-art methods. Finally, Section 5 concludes the paper.

## 2. Noise Level Estimation Based on PCA

For an input noisy image I of size M×N, the image is scanned pixel by pixel, point by point, in a sliding window fashion; y is the set of overlapping patches of size $r=d^{2}$, containing a total of s=(M−d+1)(N−d+1) patches. The matrix columns of the patches $y_{i}$ are vectorized:

(1) $$y_{i}=x_{i}+n_{i},$$

where $x_{i}$ is the i-th noiseless (original image) patch, $n_{i}$ is additive white noise following a Gaussian distribution $N\left(0,\sigma^{2}\right)$, and $y_{i}$ is the corresponding noise patch. Assuming that the noise vectors are uncorrelated with each other, refs. [27,28] used principal component analysis on this noise model to derive the following equation:

(2) $$\lambda_{\min}\left(\sum_{y}\right)=\lambda_{\min}\left(\sum_{x}\right)+\sigma^{2},$$

where $\lambda_{\min}\left(\sum_{y}\right)$ and $\lambda_{\min}\left(\sum_{x}\right)$ are the minimum eigenvalues of the covariance matrices $\sum_{y}$ and $\sum_{x}$ respectively. However, for the blind noise estimation problem, the noiseless image is unknown information [36]. It can be assumed that $\lambda_{\min}\left(\sum_{x}\right)$ of the set of patches **x** covered by weak-textured and relatively homogenous regions in the noisy image is equal to zero. Then, Equation (2) can be rewritten as:

(3) $$\sigma^{2}=\lambda_{\min}\left(\sum_{\bar{y}}\right),$$

where $\bar{y}$ is the subset of patches in set **y** that satisfy the homogenous conditions. The minimum eigenvalue of the covariance matrix $\sum_{\bar{y}}$ is equal to the actual noise level, so screening homogenous patches from the set of raw patches is a key in the noise estimation algorithm based on principal component analysis. 

An appropriate measure of image structure must be determined to select low-ranked patches with similar structures [37]. The gradient covariance matrix [38] can be used to detect image texture and structure, and Liu et al. [27] proposed an iterative weak-textured patch selection method that uses the trace of a patch’s gradient covariance matrix as a texture strength measure. The statistical properties of the texture strength ξ(n) satisfy the gamma distribution, and when the texture strength of a patch is less than the threshold τ, the patch can be considered a weak-textured patch. In the k-th iteration, the threshold τ can be expressed as a function of the given significance level δ and the noise level $\sigma_{n}^{\left(k\right)}$ of the k-th iteration as follows:

(4) $$\tau =\sigma_{n}^{\left(k\right)}{}^{2}F^{-1}\left(\delta ,\frac{d^{2}}{2},\frac{2}{d^{2}}tr\left(D_{x}{}^{T}D_{x}+D_{y}{}^{T}D_{y}\right)\right),$$

where $F^{-1}\left(\delta ,\alpha ,\beta \right)$ is the inverse gamma cumulative distribution function with shape parameter α and scale parameter β, δ is the probability that the strength of the texture is not greater than τ, $\sigma_{n}^{\left(k\right)}$ is the standard deviation of the Gaussian noise at the k-th iteration, and $D_{x}$ and $D_{y}$ represent the matrices of horizontal and vertical derivative operators.

The local variance is also an important feature for characterizing the structural information of the image. When the local variance of the patch is large, it indicates that the patch is a high-frequency part of the image, such as the edge and corner point regions. The local variance of $y_{i}$ is defined as follows:

(5) $$\sigma_{P}^{2}=\frac{1}{\left(d^{2}-1\right)}\sum_{y_{i}\in y}\left(y_{i}-\mu \right),$$

where μ is the average gray value of $y_{i}$. The $\sigma_{P}^{2}$ of the flat patch obeys a gamma distribution $\Gamma \left(r/2,r/2\sigma^{2}\right)$. Based on the nature of the gamma distribution we can obtain the mean value of the variance $E\left(\sigma_{P}^{2}\right)=\sigma^{2}$. Thus, we can use the mean local variance to estimate the noise. For the flat patch, Ping et al. [39] proved the following theorem:

**Theorem** **1.** *There must exist a unique* ${\tilde{\sigma }}^{2}\left(\tilde{\sigma }>0\right)$ *such that *

(6) $${\tilde{\sigma }}^{2}=E(\sigma_{P}^{2}|\sigma_{P}^{2}\leq \lambda {\tilde{\sigma }}^{2}),$$

(7) $${\tilde{\sigma }}^{2}=\rho \sigma^{2},$$

* with*

(8) $$\rho =\frac{\int_{0}^{F^{-1}\left(\delta ,r/2,2/r\right)}xz\left(x\right)dx}{\int_{0}^{F^{-1}\left(\delta ,r/2,2/r\right)}z\left(x\right)dx},$$

(9) $$\lambda =F^{-1}\left(\delta^{\prime },r/2,r/2\right)/\rho ,$$

* where* $z\left(x\right)=\sigma^{2}f\left(\sigma^{2}x\right)$ *represents the pdf of* Γ(r/2,r/2) *,* $F^{-1}\left(\delta ,\alpha ,\beta \right)$ *is the inverse Gamma cumulative distribution function, and* $\delta^{\prime }$ *, which is a given significance level, denotes the probability of that local variance is no more than* $\lambda {\tilde{\sigma }}^{2}$ *, i.e.,* $P\left(\sigma_{P}^{2}\leq \lambda {\tilde{\sigma }}^{2}\right)=\delta^{\prime }$ *(* P(⋅) *presents the probability).*

Following Theorem 1, we consider the patches whose local variances do not exceed $\lambda {\tilde{\sigma }}^{2}$ to be flat patches. Assuming that the patches obtained after the selection process of weak-textured and flat patches are homogenous patches, the true noise variance can be obtained by performing principal component analysis-based noise estimation on these patches. However, for images with high texture and high rank, the homogenous patch selection method has some obvious drawbacks. Patches with small local variance $\sigma_{P}$ or texture strength ξ(n) do not necessarily satisfy the homogeneity condition, and patches containing high-frequency information with high similarity are also low-rank patches [40]. Distinguishing such patches for collection is difficult, so homogenous patches will not be fully extracted when relying only on the above patch selection methods.

Most of the eigenvalues of a noisy image fluctuate around the true noise variance rather than in an absolutely equal relationship, which requires us to compute the standard deviation of the noise based on the statistical properties of the eigenvalues. The methods in [27,28] assumed that the smallest eigenvalue $\lambda_{\min}\left(\sum_{\bar{y}}\right)$ of homogenous patches is an estimate of the noise variance $\sigma_{n}^{2}$. However, experimental results have shown that the estimated noise variance is consistently smaller than the true noise variance $\sigma^{2}$.

Due to the inherent information redundancy and correlation of natural images, the patches obtained from images are usually located in low-dimensional subspaces [41]. $S={\left\{\lambda_{i}\right\}}_{i=1}^{r}$ is the set of eigenvalues of the homogenous patch set $\bar{y}$. Given $\lambda_{1}\geq \lambda_{2}\geq \ldots \geq \lambda_{r}$, we can represent $S={\left\{\lambda_{i}\right\}}_{i=1}^{r}$ as $S_{1}\cup S_{2}$, where $S_{1}={\left\{\lambda_{i}\right\}}_{i=1}^{m}$ denotes the eigenvalue of the principal dimension and $S_{2}={\left\{\lambda_{i}\right\}}_{i=m+1}^{r}$ denotes the eigenvalue of the redundant dimension [15]. While the eigenvalues of the redundant dimension ${\left\{\lambda_{i}\right\}}_{i=m+1}^{r}$ follow a Gaussian distribution $N\left(\sigma^{2},2\sigma^{4}/s\right)$, the expected value of $\lambda_{i}$ can be approximated as [42,43]:

(10) $$E\left(\lambda_{i}\right)\approx \sigma^{2}+\Phi^{-1}\left(\frac{m-r-\alpha +1-i}{m-r-2\alpha +1}\right)v,$$

where α=0.375 and Φ(x) denotes the cumulative distribution function of the standard Gaussian distribution. When the number of redundant dimensions m−r>1, we obtain the minimum eigenvalue that is intrinsically smaller than the actual noise level, $E\left(\lambda_{1}\right)<\sigma^{2}$. The result of Equation (3) underestimates the noise variance, and the accuracy of the estimation results progressively decreases as the number of redundant dimensions increases.

**Figure 1:**
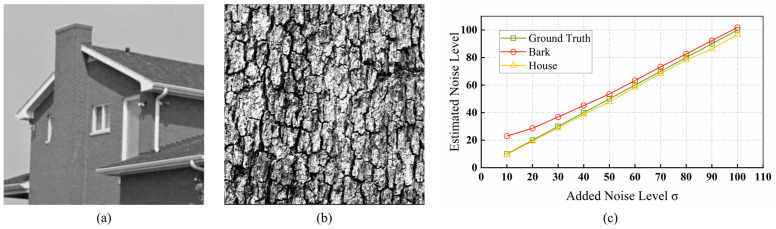
Two images with different texture strengths. (**a**) “House”, (**b**) “Bark”, and (**c**) Noise level estimation curves for the reference image.

The redundant dimension assumption also has limitations because highly textured images have extremely rich textures with large differences in pixel values. Therefore, detailed information tends to be incorrectly included in the noise signal, resulting in a minimum eigenvalue being greater than zero [44]. Figure 1 shows two images with different texture levels, “Bark” and “House”, and the results of the estimated noise standard deviation. “House” has a simpler structure, with most of the localized areas relatively smooth and having weak texture strength, and the minimum eigenvalue is slightly smaller than the actual noise level. “Bark” presents complex details with fine textures, including rich edges and ridged scenes, and the minimum eigenvalue is larger than the actual noise level, especially in low-noise conditions. Obviously, the noise estimation premised on redundancy assumptions will overestimate the noise level, which will seriously reduce the accuracy of the estimation results.

## 3. Proposed Method

### 3.1. Overall Texture Strength

**Figure 2:**
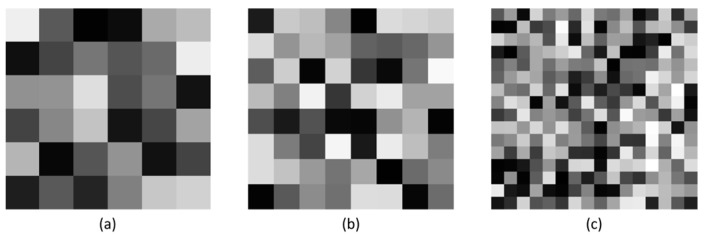
Overall texture strength for three different noise-free mosaic images. The weak-textured image has smaller values. (**a**) Δ=3.7067, (**b**) Δ=5.2795, (**c**) Δ=8.2543.

The proposed noise estimation algorithm has to avoid the influence of the noisy image’s own texture structure characteristics on the noise estimation results. We need to detect the overall texture strength of the uniform block and optimize the algorithm parameters. It is known that the local variance strongly characterizes the detailed information of the image. We define the parameters as a measure of the overall texture strength of the image, which is calculated as follows:

(11) $$\Delta ={\bar{\sigma }}_{P}-\lambda_{r},$$

where ${\bar{\sigma }}_{P}$ is the average local variance of the patches and $\lambda_{r}$ is the minimum eigenvalue of the covariance matrix. As shown in Figure 2, among the three noiseless images with different texture intensities, a smaller Δ indicates an image with a simple texture structure and Δ gradually increases as the complexity of the structure increases. This metric will be introduced as an important variable in the statistical analysis process of the feature values.

### 3.2. Deletion of Outliers

The eigenvalues obtained from the computation of the set of homogenous patches consist of the eigenvalues of the redundant dimension and the upper outliers of the main dimension. However, the number of redundant dimensions is unknown in noise estimation. We can estimate the number of redundant dimensions by removing the eigenvalues in the main dimension $S_{1}$, which represents the image information from S. We use the statistical properties of the eigenvalues of the redundant dimensions $S_{2}$ to compute the exact noise standard deviation. 

The MAD is a robust measure of sample deviation in univariate numerical data and is often used to screen out outliers in data [45]. The method primarily determines whether an item is an outlier by determining whether its deviation from the median value is within a reasonable range. This method is mainly applied in the wavelet domain, We also use this method to remove the top outliers of the set S by first calculating the MAD of the set S, and then executing the judgment and outlier removal for all elements in the set. The calculation process is shown below:

(12) $$\begin{array}{cc} MAD=median\left\{\left|\lambda_{i}-median\left\{S\right\}\right|\right\} & \left(i=1,\ldots ,r\right) \end{array},$$

(13) $$\lambda_{i}=\{\begin{array}{cc} \begin{array}{c} \begin{array}{cc} 0 & if \end{array}\lambda_{i}>S_{median}+nMAD\\ \begin{array}{cc} \lambda_{i} & if \end{array}\lambda_{i}\leq S_{median}+nMAD \end{array} & \left(i=1,\ldots ,r\right) \end{array},$$

where median{⋅} is the median, $S_{median}$ is the median of the set S, and n is the discriminant coefficient. If n is set as a constant, the accuracy and robustness of the algorithm will be poor. The discriminant coefficient n should be adaptively adjusted according to the Δ of the homogenous patch and the actual noise level σ. The optimal value of n is calculated as follows:

(14) $$n=\frac{{\left(2\sigma -\lambda_{r}\right)}^{2}-S_{median}}{MAD},$$

**Figure 3:**
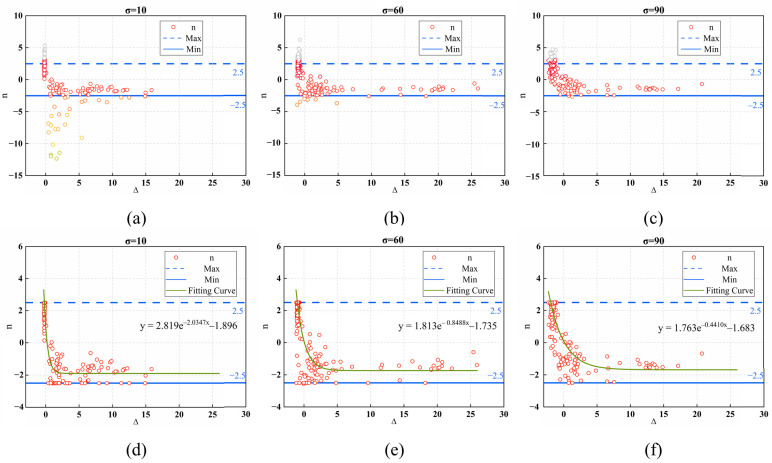
Results of Δ and n for 64 images on the Texture dataset under two noise levels. The blue solid line is the minimum allowable value of n, and the blue dashed line is the maximum allowable value of n. (**a**–**c**) Experimental results of A under different noise levels (σ=10, σ=60, and σ=90) (**d**–**f**) Corrected experimental results (σ=10, σ=60, and σ=90) and their fitted curves.

However, σ is unknown in the blind noise estimation problem. Thus, we add AWGN to images with different overall texture strengths, calculate the parameters n, Δ, and σ of the synthesized images at different noise levels, and analyze the correlation between them. Figure 3 illustrates the computational results of 188 images from three image datasets: Textures, BSDS500-val [46], and Kodak. These datasets contain a variety of scenes and textures from the real world with different overall texture intensities, which will help analyze the mapping of coefficients to parameter sums. Figure 3a–c shows that the discriminant coefficient n is in a steady state when Δ is large, but when Δ is less than 5, n has a larger range of variation, and there are many outliers in the scatter plot. This phenomenon is more obvious, especially at low noise levels. This is primarily because if an image contains highly correlated textures and structures, the image will be considered low rank and have a small Δ. At low noise levels, high-frequency signals and noise cannot be effectively distinguished, there is a large deviation between σ and $\lambda_{r}$, the value of MAD is small, and it is possible that the result n obtained by Equation (14) is an outlier, so the data need to be processed. As shown in Figure 3d–f, the range of values allowed for n is set to [−2.5, 2.5] and values outside this range are identified as outliers and replaced by the nearest endpoint values [47]. A nonlinear relationship between n and Δ can be found after data correction. The nonlinear regression model developed based on the scatterplot fitting method can be expressed as:

(15) n=aexp(−bΔ)+c

**Figure 4:**
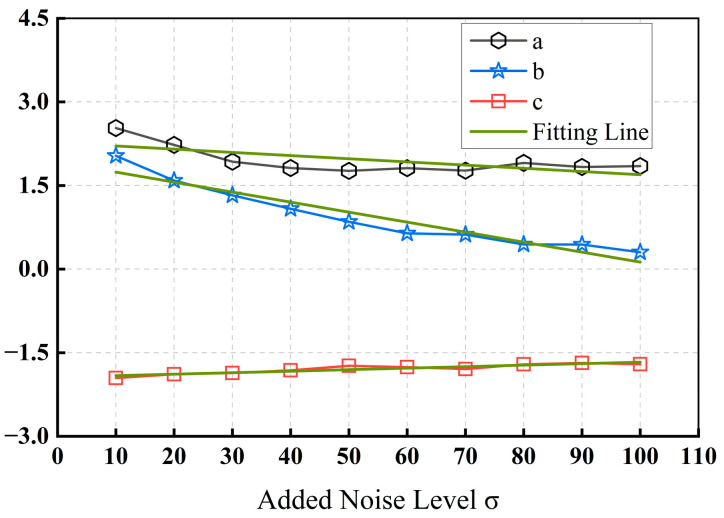
Parameters in the nonlinear regression model of *n* and Δ at different noise levels.

This nonlinear regression model is just an example for solving the problem and other regression models will also serve this purpose. Figure 3d–f demonstrates that the parameters of the regression equation change somewhat at different noise levels. To explore the relationship between the parameters a, b, and c of this nonlinear regression model and the actual noise level, we use the nonlinear regression method for n and Δ under different noise levels. The obtained model parameters are shown in Figure 4.

Parameters a and b are linearly and negatively correlated with the noise level, and both can be linearly represented by the noise standard deviation σ, while parameter c remains essentially unchanged given different noise levels. In the noise estimation process, we use $\lambda_{r}$ to replace the unknown actual noise level σ. Then, n can be established as a function with respect to Δ and $\lambda_{r}$ by rewriting Equation (15) as:

(16) $$n=\left(A_{1}\lambda_{r}+B_{1}\right)\exp\left[-\left(A_{2}\lambda_{r}+B_{2}\right)\Delta \right]+c,$$

where $A_{1}$ and $B_{1}$ denote the slope and intercept of the fitted straight line for parameter a, while $A_{2}$ and $B_{2}$ denote the slope and intercept of the fitted straight line for parameter b, respectively. Details of calculating the parameters of the mapping function are illustrated in Section 4.

### 3.3. Noise Level Estimation

After removing out-of-range outliers using dynamic thresholding, all nonzero elements in the set *S* satisfy a Gaussian distribution with a mean value of $\sigma^{2}$. Finally, the mean value of all the elements is used as the final estimate of the noise level. If the set S is the empty set, it is determined that $\lambda_{r}>\sigma$ and $\lambda_{r}$ are used as the noise estimation result, and the formula is expressed as follows:

(17) $$\sigma_{n}=\{\begin{array}{c} \begin{array}{cc} mean\left\{S\right\} & if \end{array}S\neq \varphi \\ \begin{array}{cc} \begin{array}{cc} \lambda_{r} & \end{array} & if \end{array}S=\varphi \end{array},$$

where mean{⋅} denotes the mean value. Using the mean value of the eigenvalues to estimate the standard deviation of the noise can reduce the error caused by a single eigenvalue. Based on the previous analysis, the proposed noise level estimation algorithm is summarized in Algorithm 1.
**Algorithm 1** Proposed Method**Input:** Noisy image I**Output:** Noise Level $\sigma_{n}$**Step:**1: $\sigma_{n}^{\left(0\right)}\leftarrow$ The noisy image I is divided into patches with the size of $d^{2}$.2: **for** *k* = 1 to *K* **do**3: ξ(n)← Calculate the trace of $\sum_{y}$.4: $\tau_{k}\leftarrow$ Calculate the threshold by (4).5: $\sigma_{n}^{\left(k\right)}\leftarrow$ Select the weak-textured patches.6: **end for**7: Δ← Select homogenous patches subset $\bar{y}$ by using the iterative method as described in [39].8: S← Compute the eigenvalues of $\sum_{\bar{y}}$.9: n← Calculate the discriminant coefficient by (16).10: $\sigma_{n}\leftarrow$ Remove the outliers in S.

## 4. Experimental Results

**Figure 5:**
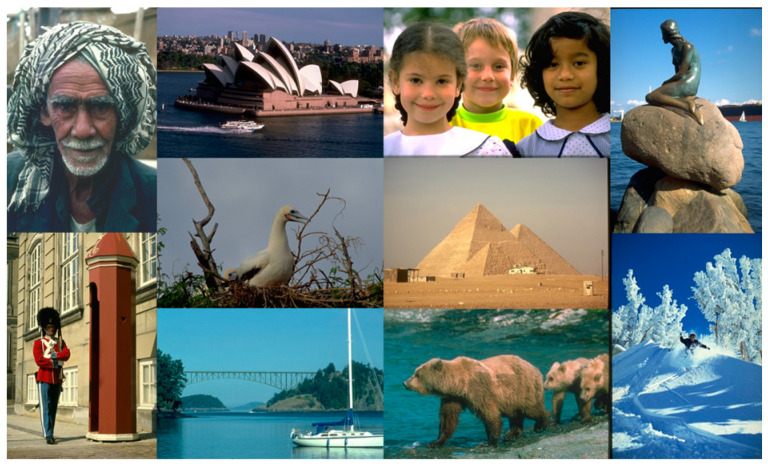
Some natural images from the BSDS500 database.

In this section, the performance results of the proposed algorithm on synthetic noisy images are obtained through experimental tests. We use the following two classical image datasets, which are commonly used to evaluate image noise estimation algorithms: the BSDS500-test dataset and the Textures dataset. The BSDS500-test dataset contains 200 real-world scenes involving scenes with diversity (e.g., transportation, architecture, landscape, nature, people, flora, and fauna), and Figure 5 shows some natural images in the BSDS500 dataset. 

**Figure 6:**
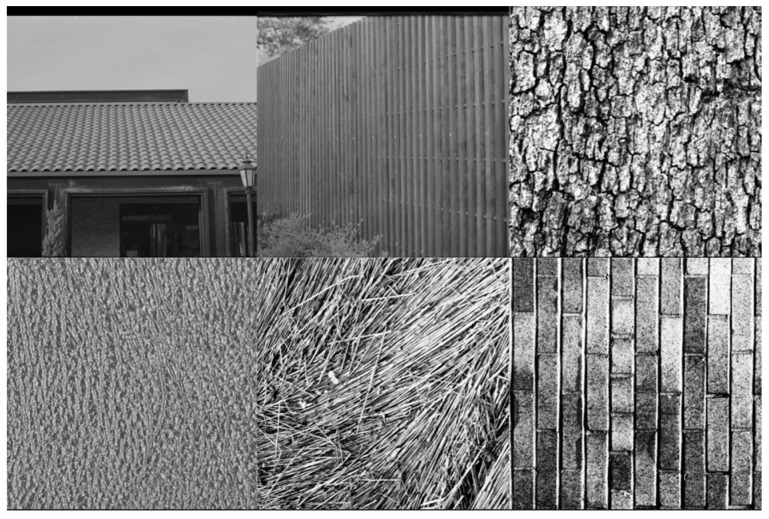
Several images from the Textures database.

The Texture dataset contains 64 texture images, including surface texture images of various materials with differences in features such as size, shape, and color, several texture images are shown in Figure 6. 

In our experiments, we added AWGN with variance σ from 10 to 100 in steps of 10 to the test images to generate synthetic images, and we ran a total of 100 simulations for each image and noise level. 

We selected five of the most commonly used and state-of-the-art noise estimation algorithms [14,27,28,29,30] for performance comparison with the proposed algorithm. In this study, the source codes of these methods are downloaded from the corresponding authors’ websites. To ensure the authenticity and reliability of the comparison results, the default parameters provided by the authors were used for all five algorithms. In the comparison experiments, the final noise level estimates were obtained by calculating the average of the noise level estimates for the different color channels while testing the color images. All algorithms were implemented on a MATLAB2021a platform (8 GB RAM, Intel(^®^) Core(™) i5-6500 CPU, 3.20 GHz processor (Intel, Santa Clara, CA, USA)).

### 4.1. Evaluation Metrics

In this study, we use the following three commonly used evaluation metrics to evaluate the performance of the proposed algorithm: bias (Bias), standard deviation (Std), and root mean square error (RMSE), which reflect the accuracy, robustness, and overall performance of the algorithm, respectively. The calculation formulas are as follows [29,40]:

(18) $$Bias\left(\hat{\sigma }\right)=E\left|\sigma -E\left(\hat{\sigma }\right)\right|,$$

(19) $$Std\left(\hat{\sigma }\right)=\sqrt{E{\left[\sigma -E\left(\hat{\sigma }\right)\right]}^{2}},$$

(20) $$RMSE\left(\hat{\sigma }\right)=\sqrt{Bias^{2}\left(\hat{\sigma }\right)+Std^{2}\left(\hat{\sigma }\right)},$$

where $\hat{\sigma }$ is the output result of the noise estimation algorithm. When these three evaluation metrics are smaller, the performance of the noise estimation algorithm is better.

### 4.2. Parameter Configuration

Before validating the proposed algorithm, the parameters in Equation (16) were obtained by sampling the image datasets. We calculated the values of parameters n, Δ, and $\lambda_{1}$ of the synthetic images at various noise levels by adding known noise levels to the reference images in the three image datasets as described in Section 3.2. The process was repeated for a total of 100 experimental simulations and the parameters in Equation (16) were calculated using multivariate nonlinear regression to obtain robust nonlinear regression model parameters. The results are presented in Table 1. 

Using the parameters obtained from the image dataset ensures the accuracy of the redundant dimension prediction and improves the accuracy and robustness of the noise estimation results. The images used to obtain the parameters of the regression model and the images used for validation are mostly mutually exclusive. 

In the proposed algorithm, there are four fixed parameters in the patch selection process. The significance level δ and the number of iterations K were used directly with the best parameters chosen in [27]. Two other important parameters of the algorithm: the significance level $\delta^{\prime }$ and the patch size d, need to be set. We first selected a range of parameters based on the default parameters of other comparative algorithms for reference and determined the optimal parameters by testing the proposed algorithm using the trial and error method. The parameter values for d ranged from 4 to 9, and the parameter values for $\delta^{\prime }$ ranged from 0.7 to 0.9. We introduced these parameters into the proposed algorithm and applied the proposed algorithm to the BSDS500-train dataset. The simulations were performed according to the experimental methodology described in Section 4, and evaluation metrics were used to ensure that the parameter settings were optimal.

**Figure 7:**
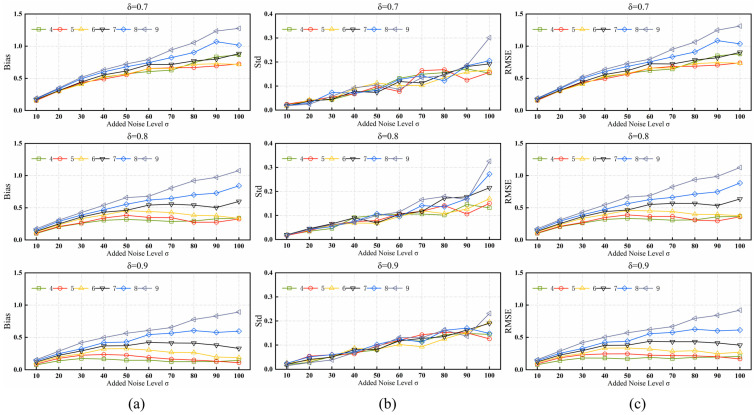
Results of the proposed algorithm with various values of parameters δ and d for the BSDS500-train dataset, (**a**) Bias, (**b**) Std, and (**c**) RMSE.

The experimental results are shown in Figure 7, which clearly shows that the performance of the proposed algorithm is extremely similar when $\delta^{\prime }$ is set to 9 and d is set to 4 or 5. The accuracy and robustness are better under these parameters than under other parameter combinations. The image characteristics, noise strength, and application-specific requirements should also be accounted for when choosing the appropriate patch size d and significance level $\delta^{\prime }$. Because smaller patches are more sensitive to structure, the results may be high and unstable under low noise levels and large structural differences, whereas larger patches contain more pixel information for statistical analysis, resulting in higher accuracy and reliability. Therefore, d is set to 5 and $\delta^{\prime }$ to 0.9 as the default parameters. The initial parameters of the proposed algorithm are listed in Table 1, this set of parameters was used for all experiments in this section.

### 4.3. Performance Comparison

To test the robustness of the algorithm to scene transformations, we conducted experiments on the BSDS500-test dataset with high complexity and realism, and Table 2, Table 3 and Table 4 illustrates the results of the accuracy, reliability, and overall performance comparisons.

The proposed algorithm is clearly superior to the other methods in all evaluation metrics and maintains a stable performance level in the selected noise range of 10 to 100. The proposed model achieves the best accuracy, robustness, and overall performance when compared with other algorithms. The results also show that our algorithm is not limited to a single situation and can effectively respond to the needs of different scenarios.

We further evaluated the performance of the algorithm on high-frequency images from the challenging Textures dataset, where most of the tests contained a large range of fine structures. As can be seen from Table 5, Table 6 and Table 7, when the noise standard deviation is 10, the Bias of [27] slightly outperforms the proposed algorithm, so there is no significant difference. The Std and RMSE [28] are slightly higher than the proposed algorithm under low noise level conditions. However, as the noise level increases, in the noise level range of 40–100, our algorithm gradually establishes a significant performance advantage, and every performance indicator is ahead of the comparison method.

The experimental results in this subsection show that our algorithm mitigates the underestimation in traditional noise estimation algorithms for images with the presence of homogenous regions, and effectively avoids the loss of image data information and the aggravation of overestimation due to redundancy assumptions in low-noise conditions for highly textured images.

### 4.4. Computational Complexity

For an image, when considering the computational complexity of Algorithm 1, we must focus on the calculation of the sample covariance matrix, as it is applied to calculate the initial noise standard deviation (Equation (3)), and two iterative computational procedures: weak texture patches search and flat patches search. Algorithm 1 generally has a computational complexity $O\left(d^{2}\times M\times N\right)$ in generating the overlapping patches, and $O\left(d^{2}\times M\times N\right)$ in calculating the eigenvalue of its covariance matrix. O(d×M×N) in searching the weak-textured patches, and $O\left(d^{2}\times M\times N\right)$ in calculating the eigenvalue of its covariance matrix. According to the explanation in [39], the total computational complexity in searching the flat patches is $O\left(Max\_step\times M^{2}\times N^{2}\right)$; obviously, the computational complexity is much larger than that of the weak texture patch search process. $O\left(d^{2}\times M\times N\right)$ in calculating the overall texture strength, $O\left(d^{3}\right)$ in calculating the redundant dimension, and $O\left(d^{2}\right)$ in calculating the final estimate of the standard deviation of actual noise level.

The most expensive part of Algorithm 1 is the flat block searching, which is much more complex than the other steps, thus it has an overall computational complexity of $O\left(Max\_step\times M^{2}\times N^{2}\right)$. (In this study Max_step=100 and d=5).

We also evaluate the computational complexity by comparing the running time in seconds of the proposed algorithm with that of other noise estimation algorithms. The average execution time of various algorithms on a 512 × 512 image is shown in Table 8.

The proposed algorithm possesses a faster running speed than [14,28,29]. Although refs. [27,30] are faster than the proposed algorithm, they perform much worse in the experiments in Section 4.3.

## 5. Conclusions

In this study, we propose a noise estimation algorithm to estimate the true noise variance from a single noisy image with complex texture and strong noise. Using the PCA noise estimation algorithm based on homogenous patches as a foundation, we discuss the relationship between eigenvalues, noise strength, and scene complexity and analyze the reasons for the generation of underestimation and overestimation. We optimize the outlier removal process by introducing the absolute median difference. According to the data sample statistics, the nonlinear regression model of discriminant coefficients is fitted to improve the accuracy of the estimation results, which makes the algorithm more robust.

To validate the performance of the proposed algorithm, we compare it with five state-of-the-art algorithms under different types of datasets and different noise levels and further validate it in denoising applications. The experimental results show that the accuracy and robustness of the proposed algorithm are the highest in most scenarios over a wide range of noise levels. In some scenarios, the algorithm also runs faster while achieving the same overall performance. In addition, the performance of the algorithm is extremely stable on different datasets, having better results for both high- and low-frequency images.

For the algorithm proposed in this study, it is necessary to build a more comprehensive noisy image dataset in the future, including more types of scenes and different levels of noise, for training and expanding the algorithm. Explore the use of different feature extraction methods, such as wavelet transform, quantum mechanics, etc., to improve the robustness and performance of the algorithm. The current nonlinear regression model and its parameters are not necessarily the optimal solution. The optimal model parameter configuration can be selected by cross-validation, optimization algorithms, and other methods to try to improve and optimize the algorithm and apply it to a wide range of fields, such as remote sensing [48] image, Channel noise [49], and so on.

In this study, the noise model of the images is assumed to be a zero-mean additive Gaussian distribution, but in real-world applications, the noise may be more complex. In addition to the common Gaussian noise model, other types of noise need to be considered, such as salt and pepper noise and Poisson noise, and understanding the nature and generation mechanism of these noise models is crucial for the expansion of the algorithm. Next, we will also look into the possibility of extending the research algorithm to multiplicative noise evaluation, and we will also aim to improve the noise parameter estimation algorithm by using more robust methods such as [50]. According to the characteristics of different noise types, we can use targeted preprocessing methods and select suitable features to extract the noise information in the image. In order to adapt to the modeling and estimation of different types of noise, we need to adjust the model parameters in the algorithm accordingly or explore the design of more flexible model structures.

## Figures and Tables

**Table 1:** Initial parameters of the algorithm.

$A_{1}$	$B_{1}$	$A_{2}$	$B_{2}$	c	d	$\delta^{\prime }$	K	δ
−0.006	2.267	−0.018	1.921	1.785	5	0.9	3	$1-10^{-6}$

**Table 2:** The Bias results of different AWGN estimation algorithms on the BSDS500-Test Dataset. The best results are highlighted in bold.

σ	[14]	[27]	[28]	[29]	[30]	Proposed
10	0.25	0.16	0.45	0.05	0.80	**0.03**
20	0.29	0.31	0.89	0.10	0.71	**0.03**
30	0.47	0.47	1.35	0.14	0.62	**0.05**
40	0.70	0.63	1.80	0.20	0.65	**0.06**
50	1.07	0.78	2.25	0.24	0.75	**0.08**
60	1.78	0.95	2.66	0.29	0.70	**0.10**
70	0.93	1.10	3.14	0.33	1.13	**0.12**
80	0.82	1.26	3.60	0.38	1.04	**0.15**
90	1.28	1.41	4.05	0.44	0.94	**0.26**
100	1.70	1.58	4.49	0.47	0.88	**0.43**
Average	0.93	0.86	2.47	0.26	0.82	**0.13**

**Table 3:** The Std results of different AWGN estimation algorithms on the BSDS500-Test Dataset. The best results are highlighted in bold.

σ	[14]	[27]	[28]	[29]	[30]	Proposed
10	0.57	0.03	0.09	0.04	0.99	**0.02**
20	0.63	0.06	0.18	0.08	0.85	**0.04**
30	0.87	0.09	0.28	0.11	0.81	**0.06**
40	1.18	0.12	0.39	0.15	0.80	**0.07**
50	2.57	0.14	0.46	0.18	0.82	**0.09**
60	2.98	0.18	0.56	0.23	0.81	**0.12**
70	1.41	0.20	0.63	0.25	1.01	**0.13**
80	1.21	0.24	0.75	0.29	1.03	**0.15**
90	1.90	0.28	0.84	0.33	1.03	**0.18**
100	2.89	0.31	0.92	0.37	1.04	**0.19**
Average	1.62	0.17	0.51	0.20	0.92	**0.10**

**Table 4:** The RMSE results of different AWGN estimation algorithms on the BSDS500-Test Dataset. The best results are highlighted in bold.

σ	[14]	[27]	[28]	[29]	[30]	Proposed
10	0.62	0.16	0.46	0.06	1.27	**0.04**
20	0.69	0.32	0.91	0.12	1.11	**0.05**
30	0.99	0.48	1.38	0.18	1.02	**0.08**
40	1.37	0.64	1.84	0.25	1.03	**0.09**
50	2.78	0.79	2.3	0.3	1.11	**0.12**
60	3.48	0.96	2.71	0.37	1.07	**0.16**
70	1.69	1.11	3.2	0.42	1.52	**0.18**
80	1.46	1.28	3.68	0.48	1.46	**0.21**
90	2.29	1.43	4.14	0.55	1.39	**0.32**
100	3.36	1.61	4.58	0.6	1.37	**0.47**
Average	1.87	0.88	2.52	0.33	1.23	**0.17**

**Table 5:** The Bias results of different AWGN estimation algorithms on the Textures Dataset. The best results are highlighted in bold.

σ	[14]	[27]	[28]	[29]	[30]	Proposed
10	3.09	**1.20**	1.40	2.15	7.66	1.24
20	2.68	1.11	**0.78**	1.38	7.26	0.88
30	2.94	0.71	**0.62**	1.00	6.83	**0.62**
40	3.29	0.53	0.60	0.80	6.62	**0.52**
50	3.60	0.50	0.86	0.68	7.08	**0.47**
60	3.97	0.47	1.07	0.57	6.76	**0.41**
70	4.18	0.49	1.47	0.54	6.58	**0.36**
80	4.71	0.52	1.69	0.50	6.11	**0.35**
90	5.02	0.57	1.99	0.46	5.58	**0.33**
100	5.09	0.63	2.38	0.46	5.28	**0.37**
Average	3.86	0.67	1.28	0.85	6.57	**0.55**

**Table 6:** The Std results of different AWGN estimation algorithms on the Textures Dataset. The best results are highlighted in bold.

σ	[14]	[27]	[28]	[29]	[30]	Proposed
10	5.18	1.62	**1.42**	2.93	7.61	1.52
20	3.76	2.68	**0.87**	1.71	7.39	1.10
30	3.84	1.58	**0.71**	1.36	7.60	0.96
40	4.01	1.02	0.82	1.09	7.82	**0.79**
50	4.34	0.89	1.10	0.97	7.90	**0.70**
60	4.76	0.68	1.26	0.78	7.75	**0.60**
70	5.13	0.69	1.60	0.80	7.25	**0.52**
80	5.75	0.66	1.74	0.74	6.96	**0.46**
90	6.56	0.64	1.77	0.70	6.61	**0.42**
100	6.71	0.62	1.96	0.65	6.43	**0.55**
Average	5.00	1.11	1.33	1.17	7.33	**0.76**

**Table 7:** The RMSE results of different AWGN estimation algorithms on the Textures Dataset. The best results are highlighted in bold.

σ	[14]	[27]	[28]	[29]	[30]	Proposed
10	6.04	2.01	1.99	3.63	10.80	**1.97**
20	4.61	2.90	**1.17**	2.19	10.36	1.41
30	4.83	1.74	**0.94**	1.69	10.22	1.14
40	5.19	1.15	0.99	1.36	10.24	**0.97**
50	5.64	1.02	1.40	1.19	10.61	**0.85**
60	6.20	0.82	1.66	0.96	10.29	**0.73**
70	6.62	0.85	2.17	0.96	9.79	**0.64**
80	7.43	0.83	2.43	0.89	9.26	**0.58**
90	8.26	0.85	2.66	0.84	8.65	**0.54**
100	8.42	0.88	3.08	0.79	8.32	**0.66**
Average	6.32	1.31	1.85	1.45	9.85	**0.95**

**Table 8:** Average Running Time of Different Noise Estimation Algorithms for 512 × 512 Images.

Method	[14]	[27]	[28]	[29]	[30]	Proposed
Time (s)	0.72	0.48	0.62	1.24	0.17	0.54

## Data Availability

The datasets used in this paper are publicly available [46] or can be obtained on the website: https://sipi.usc.edu/database/database.php?volume=textures (accessed on 13 August 2023), https://www2.eecs.berkeley.edu/Research/Projects/CS/vision/bsds (accessed on 13 August 2023), https://r0k.us/graphics/kodak/ (accessed on 13 August 2023).
